# Evidence of spontaneous cardiac-locomotor coupling during daily activities in healthy adults

**DOI:** 10.3389/fphys.2024.1394591

**Published:** 2024-08-13

**Authors:** Aurora Rosato, Matilda Larsson, Eric Rullman, Seraina A. Dual

**Affiliations:** ^1^ Department of Biomedical Engineering and Health Systems, KTH Royal Institute of Technology, Stockholm, Sweden; ^2^ Division of Clinical Physiology, Department of Laboratory Medicine, Karolinska Institute, Karolinska University Hospital Huddinge, Stockholm, Sweden

**Keywords:** synchronization, cardiovascular system, movement, wearable sensor, exercise, lifelogging

## Abstract

**Introduction:**

One way to improve exercise performance and protect heart health is the extended synchronization of the stepping with the diastolic phase of the cardiac cycle. Cardiac-locomotor coupling (CLC) happens when the step rate (SR) equals the heart rate (HR). The extent of CLC in daily life is unknown. This study aims to analyze spontaneous occurrences of CLC during daily activities.

**Methods:**

A retrospective analysis of daily life recordings from a wrist-worn sensor was undertaken (PMData, N = 16, 5 months duration). The deviation between HR and SR was used to define CLC (deviation ≤ 1%) and weak CLC (1%< deviation ≤ 10%). The occurrence and the probability of CLC during everyday life were computed from the recordings. The CLC occurrences were stratified depending on the duration and intensity of the physical activity. Finally, a Monte Carlo simulation was run to evaluate the probability of random occurrences of CLC vs. the observed recordings.

**Results:**

Participants couple for 5% and weakly couple for 35% of the observational period. The ratio of 1:1 between HR and SR is the dominating occurrence across the study population and this overrepresentation is significant. CLC occurs mostly for long activities. The extent of CLC for various intensities of activity is subject-dependent. The results suggest that CLC is feasible for most people.

**Conclusions:**

CLC occurs spontaneously during unsupervised daily activity in everyone in our cohort, which suggests a mechanistic interaction between the cardiac and the locomotor systems. This interaction should be investigated for medical rehabilitation and sports applications in the future.

## 1 Introduction

Promoting higher muscle perfusion is key to performing well in sports and to ease the strain in patients with heart deficiencies. Actively synchronizing the stepping with the heart cycle, i.e., cardiac-locomotor coupling (CLC), has been proposed as a valid strategy to enhance muscle perfusion and blood flow, therefore leading to better physical capacity (Takeuchi et al., 2015; Constantini et al., 2018).

**FIGURE 1:**
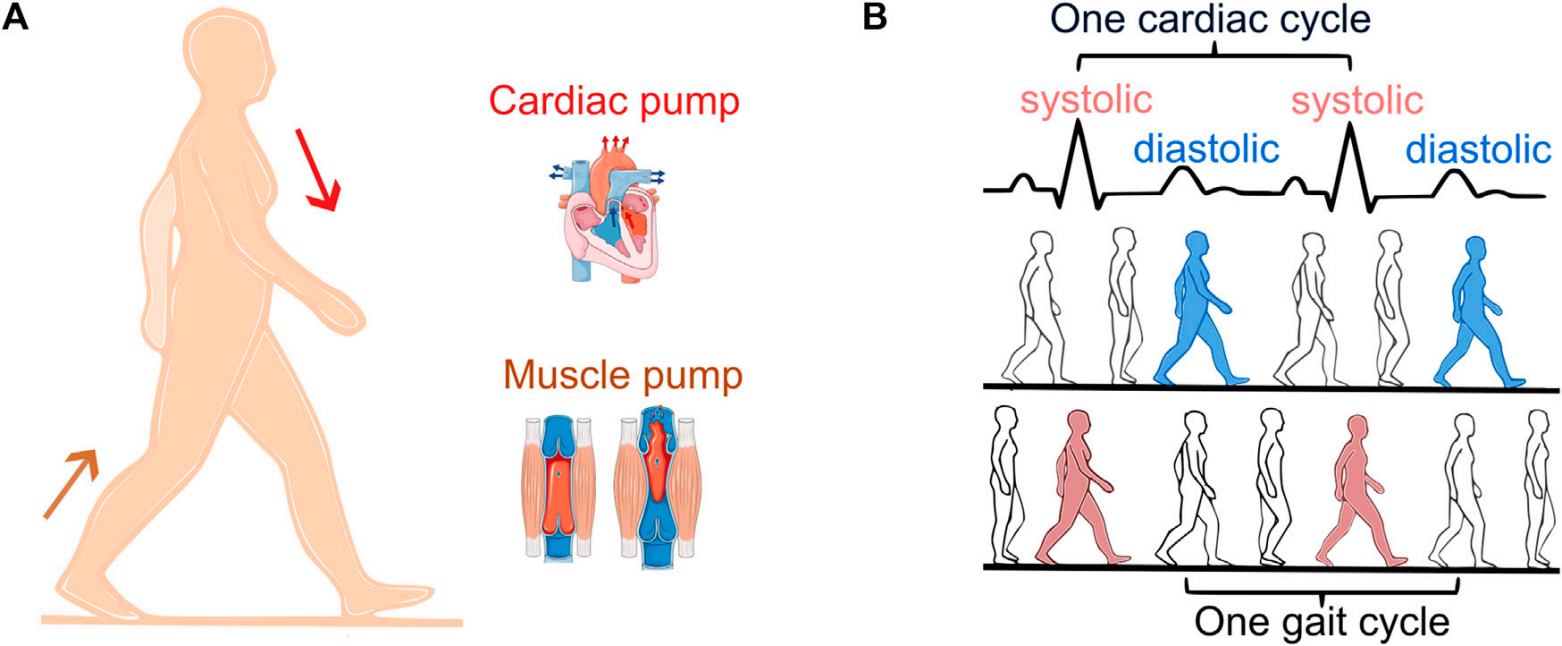
Cardiac and skeletal muscle pump **(A)**. Diastolic (synchronization out of phase, blue) vs. systolic stepping (synchronization in phase, red) **(B)**. One cardiac cycle is defined as the time interval between two R-peaks of the electrocardiogram (black).

During locomotion, the total blood flow, i.e., cardiac output is affected by the cardiovascular, respiratory, and locomotor systems. The pumping of the heart delivers blood to the contracting muscles and the skeletal muscle pump, through periodic increases in intramuscular pressure, enhances the venous return of the blood to the heart (Figure 1A) (Novak et al., 2007). CLC occurs if these two pumps become entrained with equal contraction rates meaning that the cadence of the exercising limb is the same as the heart rate (HR) (Figure 1B).

Synchronization is commonly defined as the alignment of rhythms observed by a constant ratio between their periods. CLC is observed by a 1:1 ratio between the heart cycle and the gait cycle. In addition, phase synchronization indicates at which point within the cardiac cycle each step occurs, defining diastolic stepping (Figure 1B, blue) vs. systolic stepping (Figure 1B, red). Hemodynamic benefits of CLC occur in diastolic stepping (O Rourke et al., 1993). One study examining the phase of spontaneous CLC found that diastolic stepping is more common, suggesting that this could enhance venous return and optimize blood flow to the exercising muscle (Niizeki et al., 1996).

The physiological advantages of diastolic stepping have been investigated in previous laboratory studies. Healthy runners showed lower systolic left ventricular pressure and higher diastolic pressures consequently in diastolic compared with systolic stepping (O Rourke et al., 1993). This pressure modulation during diastolic stepping was found to be comparable to the effect of the intra-aortic balloon pump (IABP) therapy (O Rourke et al., 1993). IABP uses counter-pulsation to increase myocardial oxygen perfusion and reduce afterload, resulting in increased cardiac output. Diastolic running also resulted in a decrease in HR and ventilation (Nomura et al., 2006; Constantini et al., 2018). In contrast, when muscle contraction was synchronized to systole, the HR increased (Niizeki et al., 1996; Niizeki and Miyamoto, 1999; Nomura et al., 2006; Constantini et al., 2018). Enhancements in exercise performance during CLC walking have also been suggested in terms of increased lower leg muscle perfusion (Takeuchi et al., 2015). Researchers showed that in patients with heart deficiencies, the stroke volume increases during diastolic vs. systolic stepping (Wakeham et al., 2023), demonstrating therapeutic potential for CLC application in the medical field as well as in sports.

Despite the promising benefits of CLC, its spontaneous occurrence and applicability in everyday contexts remain understudied. Clearly, the benefits of CLC are pronounced when more time is spent in or near CLC. An open challenge in the field is therefore whether and how CLC can be applied in everyday contexts. However, thus far, the spontaneous interaction between cardiac and locomotor systems in humans has been documented and proven only in laboratory settings (Kirby et al., 1989; Hausdorff et al., 1992; Takeuchi et al., 2022a; Novak et al., 2007; Constantini et al., 2018; Carvalho et al., 2021), although treadmills can affect a person’s natural running pattern. One recent study successfully demonstrated spontaneous CLC during short sequences of outdoor running (Perry et al., 2020). Building on this finding, our study explores CLC over a longer period. The physiological occurrence of CLC remains elusive and we do not know if humans in their daily lives experience CLC. CLC is, however, a necessary condition to allow for diastolic synchronization, and thus higher physical capacity. Studying CLC occurrence during our daily lives will increase our understanding of the presence of spontaneous interaction between the cardiac and locomotor systems and how feasibly we can optimize the interaction for endurance performances or cardiac rehabilitation.

The aim of this study is to use real-word data to study the prevalence of CLC occurrences during unsupervised daily activities in healthy adults, collected retrospectively from a smartwatch. Despite the limitations in accuracy of wrist-worn devices, the ability of smartwatches to continuously monitor over prolonged periods and during a variety of daily activities provides a powerful starting point to assess spontaneous CLC. Our hypothesis is that CLC is a common occurrence across all individuals and that each subject experiences CLC at different intensities and durations of the training activity.

## 2 Methods

### 2.1 Data set

We used the retrospectively acquired dataset PMData (Thambawita et al., 2020). The 16 healthy subjects (13 men and 3 women) aged between 23 and 60 years (29 (16.75) years) participated in the study. The time for a 5 km run and the daily resting HR were available to assess participant’s training levels. All participants have been informed about the collection and publication of the data and consented to be enrolled in the study (Thambawita et al., 2020).

The study protocol lasted 5 months, from November 2019 to the end of March 2020. During this time, the participants were encouraged to wear a smartwatch Fitbit Versa 2 (Fitbit.Inc., San Francisco) as much as possible. The smartwatch acquired SR every minute and HR every 5 s. The study organizers did not impose any restrictions or requirements on the type or duration of exercise to perform (Thambawita et al., 2020).

### 2.2 Data analysis

The data were processed using custom code in MATLAB R2022a (The MathWorks, Natick, MA) using the Statistic and Machine Learning Toolbox. The HR was averaged per minute, to match the time resolution of the SR and to account for possible fluctuation. HR and SR were synchronized via the timestamp information. To focus on instances of physical activity and avoid the inclusion of instances of sleep and rest, all data below 60 steps/min were excluded from the analysis. The threshold of 60 steps/min was previously identified as slow walking in a study population of adults older than 20 years of age (Tudor-Locke et al., 2011).

#### 2.2.1 Cardiac-locomotor coupling definition

To find occurrence of coupling, the ratio R between SR and HR (Algorithm 1) was computed for each instance.

$$R=\frac{\text{SR}\left(\frac{\text{steps}}{\min}\right)}{\text{HR}\left(\frac{\text{beats}}{\min}\right)}$$

Algorithm 1 Ratio R between SR and HR used to define coupling.

CLC is defined as a deviation between SR and HR of ≤1% (Kirby et al., 1989). We grouped the data depending on CLC strength into no CLC (deviation > 10%), weak CLC (1% < deviation ≤ 10%), and CLC (deviation ≤ 1%) for further consideration.

We used the 1%–10% range to indicate the potential feasibility of coupling. At 60 beats/min this would indicate a deviation of a maximum of 6 steps/min, whereas at 120 beats/min this would indicate a deviation of a maximum of 12 steps/min. Based on the literature, step rates can be altered up to ± 10% from the preferred running SR (Heiderscheit et al., 2011; Kiernan et al., 2024) without harmful effects, such that the 10% threshold seems an appropriate choice.

#### 2.2.2 Probability of cardiac-locomotor coupling

As HR and SR are known to be physiologically correlated, observation of CLC may be attributed to chance. Thus, we compared the observed R to a randomly simulated R_sim_ which we computed using a Monte Carlo approach for each subject. Monte Carlo simulations are used to predict the probability of a model output based on the random combination of statistically distributed input variables. In our study, the model is equivalent to the R calculation (Algorithm 1), which was fed with a random combination of the recorded values of HR (size = N) and SR (size = N) for each subject as input variables of the model. The output of the Monte Carlo simulation consists of NxN randomly computed values of R_sim_. The output of the Monte Carlo thus describes the probability distribution of all the possible R based on the measured HRs and SRs.

#### 2.2.3 Intensity of the activity

We used the metabolic equivalent of task (METs) to evaluate the intensity of physical activity. One MET represents the oxygen consumption while sitting at rest (1 MET = 3.5 mL O_2_/kg/min) (Jetté et al., 1990). Computing the SR information, we classified each instance according to the level of intensity, using the heuristic cadence thresholds of 60 steps/min, 100 steps/min, and 130 steps/min, which are associated with light, moderate, and vigorous activity (Tudor-Locke et al., 2019).

#### 2.2.4 Duration and time of the activity

We quantified the timing and duration of each activity based on the SR and HR information. An activity is defined as consecutive observations with a time difference between them smaller than 1 hour. In this way, we calculated the mean duration of activities and the time of the day of the activity.

#### 2.2.5 Subject grouping depending on coupling pattern

We grouped the subjects depending on the CLC occurrence in moderate vs. vigorous activity (Algorithm 2). We defined three groups: one with most CLC during vigorous activity (D ≤ −1.5), one with most CLC in moderate activity (D ≥ 1.5) and one with a similar extent of CLC in vigorous and moderate activity (−1.5 < D < 1.5).

$$D={\left(\text{CLC}\;\text{occurrence}\right)}_{\text{moderate}}-{\left(\text{CLC}\;\text{occurrence}\right)}_{\text{vigorous}}$$

Algorithm 2 Differences between CLC occurrence in moderate vs. vigorous activity intensity, used to define three groups.

### 2.3 Statistical analysis

A Chi-squared test was used to determine if the distribution of R, HR and SR were normal. Since the data was not normally distributed, we used non-parametric statistical tests to assess differences. Median and interquartile ranges were computed for each parameter of interest. Differences in SR and HR between CLC, weak CLC and no CLC were not controlled for intensity but calculated as a median over all intensities.

The Friedman test was instead used to assess statistically significant differences among the subjects during different activities and CLC conditions. Pearson correlation coefficient was calculated for regression analyses.

A two-sample Kolmogorov-Smirnov test was applied to test if the observed R and random R_sim_ distributions were different. A Wilcoxon signed-rank test was used to compare the percentages of CLC probabilities, the interquartile ranges and the skewness of the random vs. observed distributions.

For paired samples, the Wilcoxon signed-rank test was also used to compute the effect size $\left(r\right)$ of the results presented by dividing the standardized Z-score by the square root of the number of pairs (N = 16) as follows:

$$r=\frac{Z}{\surd N}$$

Algorithm 3 Effect size calculation with Wilcoxon signed-rank test.

Despite our dataset is not appropriate for an in-depth age analysis, we tested statistically significant differences between age groups with Kruskal-Wallis. The effect size $\left(\eta ²\right)$ was calculated by dividing the tested H statistic by the total sample size (N = 16) as follows:

$$\eta ²=\frac{H}{N-1}$$

Algorithm 4 Effect size calculation with Kruskal-Wallis.

Values presented are median (inter-quartile range), unless otherwise stated. The results were considered statistically significant at *P* ≤ 0.05.

## 3 Results

### 3.1 Subject characteristics

A total of 20’991’392 HR and 15’34’705 SR values were acquired. Demographic and physiologic data are presented in Table 1.

**TABLE 1:** Demographic characteristics of the participants, resting HR (beats/min), R, CLC percentage occurrence and pearson correlation coefficient for the entire study period (5 months). Data reported as median (inter-quartile range). HR: Heart rate, R: Coupling ratio, Algorithm 1, CLC: Cardiac-locomotor coupling.

Subject	Age	Height (cm)	Gender	Resting HR (beats/min)	R	CLC occurrence (%)	Pearson correlation coefficient
1	48	195	Male	52 (1.8)	0.96 (0.27)	3.98	0.61
2	60	180	Male	47 (1.9)	1.00 (0.23)	5.75	0.60
3	25	184	Male	56 (2.6)	0.92 (0.21)	5.30	0.53
4	26	163	Female	70 (4.5)	0.93 (0.25)	7.02	0.73
5	35	176	Male	63 (5.9)	0.87 (0.25)	3.95	0.40
6	42	179	Male	51 (2.6)	0.99 (0.33)	3.38	0.76
7	26	177	Male	58 (2.8)	0.99 (0.27)	3.99	0.77
8	27	186	Male	55 (3.1)	0.96 (0.29)	3.47	0.67
9	26	180	Male	61 (4.3)	0.98 (0.29)	3.76	0.38
10	38	179	Female	68 (3.2)	0.92 (0.25)	5.85	0.61
11	25	171	Female	64 (2.8)	0.90 (0.26)	5.43	0.52
12	27	178	Male	64 (2.8)	0.94 (0.25)	5.35	0.60
13	31	183	Male	64 (2.8)	0.86 (0.29)	3.31	0.53
14	45	181	Male	64 (4.8)	1.04 (0.27)	4.39	0.68
15	54	180	Male	54 (2.4)	0.95 (0.33)	3.06	0.32
16	23	182	Male	67 (2.2)	0.91 (0.21)	4.70	0.26

The median resting HR during the observational period suggests differences in the training level of the subjects (62.12 (8.88) beats/min). Furthermore, the time spent to run 5 km (28(8) min) differs across subjects, ranging from 18 min (well-trained) to more than 30 min (intermediate or beginner).

### 3.2 Cardiac-locomotor coupling

**FIGURE 2:**
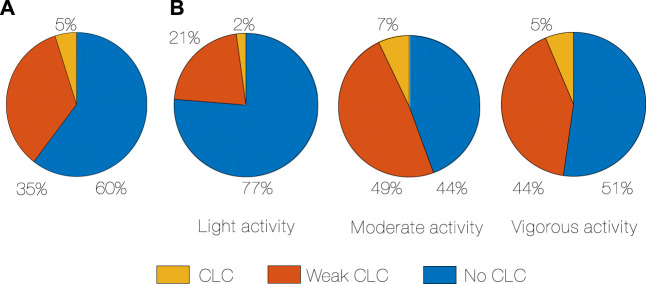
CLC occurrence in percentage of time, median for all subjects **(A)**, CLC occurrence in percentage of time during different activity intensity, median for all subjects **(B)**. CLC: Cardiac-locomotor coupling.

Evidence of CLC was found in every subject. Total CLC occurrence ranged from 3% to 7% (median 5%) and weak CLC from 26% to 40% (median 35%), across subjects (Figure 2A).

We further assessed the amount of CLC depending on the intensity of the activity (Figure 2B). The least CLC occurs during light activity, while the most CLC occurs during moderate (2% vs. 7%, *p* < 0.001, *r* = 0.88, Algorithm 3). Weak CLC also occurs more in moderate compared to light activity (49% vs. 21%, *p* < 0.001, r = 0.88). Even if there is a subgroup that has more CLC in vigorous (Algorithm 2), CLC seems to not increase directly and proportionally with an increase in physical activity.

We found higher SR in CLC vs. no CLC (109 (17) vs. 85 (23) steps/min, *p* < 0.001, r = 0.88), and in weak CLC vs. no CLC (107 (20) vs. 85 (23) steps/min, *p* < 0.05, r = 0.88).

HR was higher in CLC compared to no CLC (109 (17) vs. 101 (22) beats/min, *p* < 0.001, r = 0.67). Across subjects, the median of the R ranges between 0.87 and 1.04 (Table 1).

**FIGURE 3:**
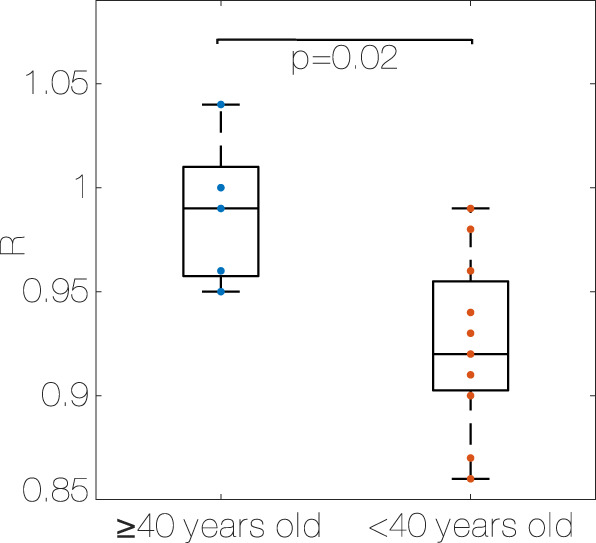
Median R for each subject, older group (≥40 years old) vs. young group (<40 years old). R: Coupling ratio, Algorithm 1.

The median R (Figure 3) is higher in the older group compared to the young one (0.99 (0.04) vs. 0.92 (0.05), *p* < 0.05, η^2^ = 0.36, Algorithm 4, older group (≥40) and young group (<40)).

In every subject, HR and SR are positively correlated (*p* < 0.001), and the Pearson correlation coefficient ranges from 0.26 to 0.77 depending on the subject. Even if not significant, the correlation coefficient is higher in the older group compared to the young one (0.61 (0.09) vs. 0.53 (0.19), older group (≥40) and young group (<40)).

### 3.3 Probability of cardiac-locomotor coupling

**FIGURE 4:**
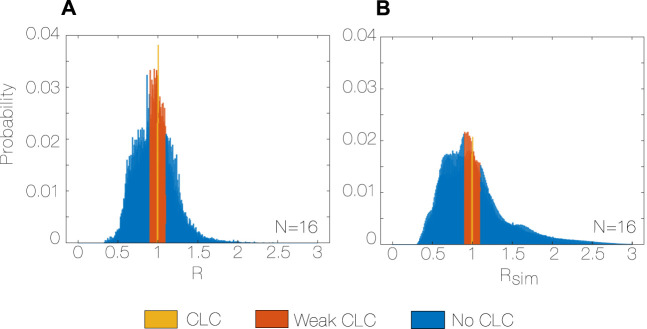
Observed R distribution with bin size = 1%, N = 16 **(A)**, and random R_sim_ distribution with bin size = 1%, N = 16 **(B)** during the whole study period (5 months). R: Coupling ratio, Algorithm 1, R_sim_: simulated coupling ratio, N: total number of subjects.

The observed distribution (Figure 4A) shows a peak at R = 0.99–1.00 (0.06), and probability of the peak R of 2.6% (0.01). The distribution is not symmetric, showing a tendency to SRs lower than HRs (skewness 0.28 (0.54)).

The observed and random distributions of R and R_sim_ are different (*p* < 0.001 for every subject, Figure 4B). In particular, the observed distribution has a median closer to the unity, it is more symmetric (skewness observed vs. random, 0.280 vs. 0.825, *p* < 0.001, r = 0.99) and less spread (interquartile range observed vs. random, 0.261 vs. 0.381, *p* < 0.001, r = 0.99) than the random distribution. Moreover, the probability of both CLC and weak CLC is 33% higher for the observed distribution (0.40 vs. 0.27, *p* < 0.001, r = 0.99) compared to the random.

### 3.4 Intensity of the activity

Subjects spent most time in moderate activity during the 5 months. The percentage of time spent in light, moderate and vigorous activity is 25% (9.5), 59% (26), and 11% (16), respectively. The extent of moderate activity is higher than the extent of vigorous activity during everyday activities (59% vs. 11%, *p* < 0.001, r = 0.83).

**FIGURE 5:**
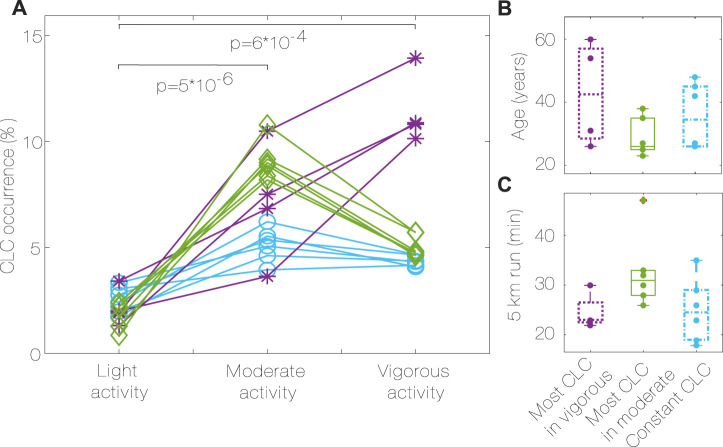
CLC occurrence during different activity intensity **(A)**, groups characteristics **(B,C)**. Most CLC in vigorous (purple, *, D ≤ −1.5), most CLC in moderate (green, ♢, D ≥ 1.5), constant CLC (blue, ○, −1.5 < D < 1.5), Algorithm 2. *P* values for differences between activities for all subjects. CLC: Cardiac-locomotor coupling, D: Difference between CLC occurrence in moderate vs. vigorous activity.

The CLC occurrence (Figure 5A) is higher when performing moderate activities compared to light activities (6.84% vs. 2.10%, *p* < 0.001, r = 0.88) and when performing vigorous activities compared to light activities (4.75% vs. 2.10%, *p* < 0.001, r = 0.88). There was no significant difference between the extent of CLC during moderate activities compared to vigorous activities. Figure 5A shows that there are three recognizable groups, depending on the difference between CLC occurrence in moderate and vigorous activity intensity.

Group 1 has the most CLC occurrence during vigorous activity, group 2 has the most CLC occurrence during moderate activity, while group 3 has similar amounts of CLC in both conditions.

The group characteristics are summarized in Table 2. No significant differences were found in age, 5 km run time, height and resting HR between the groups. Even if not significant, it can be noticed that group 1 has the oldest subjects (Figure 5B), group 3 has subjects with the lowest resting HR, the highest percentage of vigorous activity and the tallest, group 2 has the youngest subjects, with the highest resting HR, less vigorous activity, and the longest 5 km run time (Table 2; Figure 5C).

**TABLE 2:** Characteristics for the different groups depending on CLC pattern (Algorithm 2). Most CLC in vigorous (D ≤ −1.5), most CLC in moderate (D ≥ 1.5), constant CLC (−1.5 < D < 1.5). CLC: Cardiac-locomotor coupling, HR: Heart rate, R: Coupling ratio.

Variables	Most CLC in vigorous	Most CLC in moderate	Constant CLC
Age, years	42.5 (25.7)	26 (8)	34.5 (18)
Height, cm	180 (5)	178.5 (4.75)	180.5 (5.5)
Resting HR, beats/min	59 (13)	64 (2.6)	56.6 (7.4)
Time for 5 km, min	23 (2)	31 (4.2)	24.5 (8.5)
Percentage of vigorous activity, %	7.5 (9.2)	2.71 (2.93)	16.7 (18.5)
R	0.94 (0.05)	0.92 (0.017)	0.99 (0.025)
Percentage of CLC %	4.5 (2.8)	5.3 (0.56)	3.87 (0.4)
Number of instances	11,154 (6,447)	11,204 (3,029)	7,568 (3,130)

### 3.5 Duration and time of the activity

**FIGURE 6:**
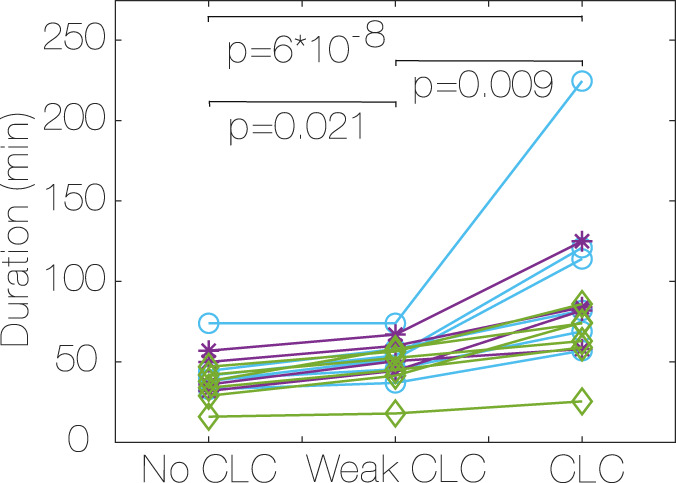
Median duration of activity for each subject, depending on CLC strength (no CLC, weak CLC and CLC). Most CLC in vigorous (purple, *, D ≤ −1.5), most CLC in moderate (green, ♢, D ≥ 1.5), constant CLC (blue, ○, −1.5 < D < 1.5), Algorithm 2. *P* values for differences between CLC strength for all subjects. CLC: Cardiac-locomotor coupling, D: Difference between CLC occurrence in moderate vs. vigorous activity.

The median duration of activities (Figure 6) is higher in CLC occurrence vs. no CLC (78.25 min vs. 38.5 min, *p* < 0.001, r = 0.88) in CLC vs. weak CLC (78.25 min vs. 52.25 min, *p* < 0.05, r = 0.88) and in weak CLC vs. no CLC (52.25 min vs. 38.5 min, *p* < 0.05, r = 0.85). Most of the activity is distributed between 10:00 a.m and 7:00 p.m. We found no correlation between the time of the day at which the activity is performed and the CLC strength (no CLC, weak CLC and CLC).

## 4 Discussion

The main finding of this study is that every subject presented CLC occurrences during daily activities and that the SR to HR ratio of 1:1 across all subjects is more common than any other ratio. Moreover, the amount of CLC observed is higher than what would be expected from random correlation of SR and HR. This result suggests that, in a physically active population with varying levels of physical fitness and age, CLC is a spontaneous and common phenomenon during daily activities. In this way, our study points towards feasibility of achieving CLC in everyday activities.

The SRs and HRs in our study are naturally positively correlated. In fact, when exercising, the cardiovascular system adapts to meet the metabolic demand of the systemic system, including the skeletal muscles (Murphy et al., 2011). The interaction between the cardiovascular and locomotor systems is affected by the parasympathetic and sympathetic systems. During exercise, the sympathetic nervous system regulates the blood supply of the exercising muscle by increasing arterial blood pressure and HR (Murphy et al., 2011). In this way, we expect the HR to increase with increased metabolic activity intensity, equivalent to an increased SR. Since SR and HR are naturally correlated in our data set, one could argue that the 1:1 is merely due to chance.

Investigation of CLC due to chance has previously been attempted using a surrogate data technique in a laboratory study (n = 10, healthy men) and they were able to show spontaneous entrainment between the cardiac and locomotor rhythm (Nomura et al., 2001). In our study, we randomly combined HR and SR from our time series with a Montecarlo approach to determine if the found behavior in the R comes from a deterministic or stochastic interaction between the two. In agreement with the results found in the laboratory setting (Nomura et al., 2001), we were able to show that in the random data, the CLC probability is 33% lower and the distribution is different compared with the observed data. This finding supports the hypothesis that CLC does not only occur by chance, but rather that is due to an entrainment of the cardiovascular and locomotor systems.

Our results that coupling occurs to varying degrees in all subjects are in contrast to studies performed in laboratory settings where some of the subjects did not express CLC (Kirby et al., 1989; Hausdorff et al., 1992; Novak et al., 2007; De Bartolo et al., 2021). One of the first laboratory studies in which spontaneous CLC was investigated, found that CLC occurred in 18/25 healthy, trained subjects for SRs between 106 and 150 steps/min (Kirby et al., 1989). During daily activities, we observed CLC in a wider range of SR (between 60 and 180 steps/min, depending on the subject) and found CLC in all subjects. This finding opens possibilities for medical application of CLC, other than sport-related applications, including people who are not elite runners or who cannot walk at high SRs. The use of a treadmill in laboratory settings comes with a limited observational period and might affect the choice of a comfortable walking speed and SR, increasing the risk of no CLC.

Weak CLC is of interest because it indicates the feasibility of actively encouraging CLC for improved training or rehabilitation effects in daily activities. While previous studies focused only on coupling, we also investigated when and to what extent the two rhythms were within 10% of each other (weak CLC). Even though CLC is present only in 5% of the observational period, HR and SR are within 10% of each other for a longer time, corresponding to 35% of the observational period. This finding supports the hypothesis that the locomotor and cardiovascular systems are naturally close to coupling, which can be achieved with only small adjustments in cadence during a wide range of exercise intensities and in a large portion of the population. In this way, CLC can be reached without changing or forcing the physiology of our body. Despite the potential clinical benefits, moving from weak CLC to CLC may prove challenging. First, changing the walking frequency by 6–15 steps/min could impact the metabolic cost of walking (Bunc, 2022). Further investigations should address potential trade-offs between CLC and the optimal metabolic cost of walking. Moreover, modulating gait might lead to unnatural walking patterns and unnatural running gait biomechanical factors, increasing risks of injuries, and inefficient or acute joint loads (Vannatta et al., 2020). Strategies for adapting the walking pattern, such as modifying the cadence or stride length, vary among individuals and have different effects on the biomechanics (Ardestani et al., 2016). Modulating cadence might also be used for mobility improvement, especially for stroke patients and injury prevention (Nijs et al., 2020). Gait modification is not the only possible strategy for CLC to happen since the HR can be adjusted to match the most comfortable SR by warming up before the session or walking on inclined surfaces. While matching SR and HR can seem artificial and unnatural, with this study we want to highlight the feasibility and potential of CLC on a wider range of walking speeds and conditions.

CLC has previously been associated with age (Novak et al., 2007), showing that CLC between SR and HR was mostly relevant for elderly subjects (70.3 ± 5.1 years) and less for young subjects (29 ± 5.1 years). In agreement with the study, we found that the median R was different depending on age, showing that elderly subjects are spontaneously closer to CLC during everyday life. Moreover, in our analysis, age is an indicator of the activity intensity at which CLC is more likely to occur. Given these age-dependent differences, it has been speculated that aging emphasizes the contribution of muscular pumping due to baroreflex downregulation (Novak et al., 2007). If so, walking at normal speed may have greater beneficial effects on cardiovascular performance in the older population at risk of cardiovascular disease. This result is supported by a recent study in which they demonstrated stronger CLC in patients with cardiorespiratory impairment (Takeuchi and Nishida, 2023) compared to healthy controls, suggesting that CLC might be a compensatory phenomenon for exercise maintenance. The clinical implications of CLC in terms of beat-to-beat pressure modulation and cardiovascular performance have still to be addressed in a healthy and elderly population, but an interesting clinically relevant potential lies behind these discoveries.

We analyzed CLC occurrence at different levels of activity intensity and correlated them to the training level of each subject. The intensity is directly related to the oxygen consumption necessary to complete the activity, thus influencing the metabolic demand. We found that, even if on average there is more CLC during moderate activities, this is subject-dependent. This study only includes a small number of subjects, limiting the interpretation of group analyses to trends. We found that the group with the most CLC in moderate activity had a higher resting HR, higher time for the 5 km run, and lower level of vigorous activity, which could suggest lower fitness. This result emphasizes how CLC is easily reached during moderate activity even for not trained subjects. Associations between exercise capacity levels and CLC in young subjects have previously been studied and it has been suggested that people with a low cardiopulmonary function and sedentary life have stronger coupling between cardiac and locomotor rhythms during exercise (Takeuchi et al., 2022b). Our dataset lacks information about exercise capacity for a complete analysis, but our findings confirm the trend that spontaneous CLC occurs to a higher extent for not trained and young subjects during moderate activity. For more trained subjects, a more detailed analysis would be necessary to uncover the key parameters in the HR and SR interaction.

Smartwatches are a powerful tool to obtain insights and information about unsupervised daily activities, that would otherwise be difficult to obtain. However, the advantage of unsupervised data collection can make it difficult to control artifacts in the data. HR detection from wrist-worn devices relies on photoplethysmography (PPG) technique, which has various limitations compared to the chest-worn sensors, which rely directly on the electrocardiogram (ECG) (Boudreaux et al., 2018).

In order to validate our results, we checked for bias in SR and HR reported by prior studies using the Fitbit Versa smartwatch which include a reference measurement for SR or HR. SR detection using Fitbit is generally underestimated across all walking speeds with an error greater than −3% in half the time (Feehan et al., 2018). Half of the measurements fall within our tolerance range ± 10% of weak coupling. In our analysis, the R distribution is left-skewed, showing a general trend of lower SR compared to HR. If the step counts were higher, we would more likely observe an R value closer to the median, which represents the CLC condition. For HR measurement, some studies reported no systematic bias (Sjöberg et al., 2021) or very little overestimation of 1.13 ± 0.83 bpm (Chevance et al., 2022). The reported mean absolute percentage errors for the smartwatch employed in this study are 11.6% ± 8.7% (Jagim et al., 2021) and 10.56% (Sjöberg et al., 2021), with a coefficient of variation ranging from 4.1% to 19% (Düking et al., 2020), depending on the intensity of the activity (overall average deviation of 11.4 bpm (Düking et al., 2020)). The just presented results show that the HR measures are only affected by random variability.

In future studies, HR and SR should be acquired by using chest-worn devices with higher accuracy in measuring gait and ECG parameters. Moreover, PPG allows for HR, but not direct R-peak detection, making it suitable for SR:HR synchronization detection, but not for phase synchronization. Considering the beneficial effects of diastolic stepping as opposed to systolic stepping, a more in-depth analysis of timings of CLC including electromyograms (EMGs) and foot switches should be considered for future studies. In this way, it would be possible to further explore CLC in everyday life and understand if the subjects synchronized each step with the diastolic or systolic part of the cardiac cycle.

## 5 Conclusion

This study introduces a new perspective on CLC applications by analyzing CLC during daily activities. CLC occurs spontaneously during unsupervised daily activity in every subject in our cohort. Subjects experience more CLC when engaging in long activities, while the preferred level of intensity is subject-specific. Moreover, the SR to HR ratio of 1:1 prevails over any other R, which could not be explained by chance. Our results further support a mechanistic interaction between the cardiac and the locomotor system and point toward the feasibility of actively supporting CLC in long activities.

### 5.1 Perspective

As presented in previous research, CLC has the potential to increase physical capacity and it could be a predominant factor in improving or hindering endurance and exercise tolerance during sport and in people with reduced heart function. However, it is currently not clear whether it is related to some spontaneous mechanism and how feasible it is in a heterogeneous population. In this study, we have shown that every subject in our cohort experiences CLC during everyday activities, with different extents and at different activity intensities. By providing evidence of CLC in daily life, this work supports further research on customized training and rehabilitation programs exploiting CLC at home.

## Data Availability

Publicly available datasets were analyzed in this study. This data can be found here: https://osf.io/vx4bk/. The dataset is also available at this link https://datasets.simula.no/pmdata/, doi 10.17605/OSF.IO/VX4BK.

## Abbreviations

CLC, Cardiac-locomotor coupling; ECG, Electrocardiogram; EMG, Electromyogram; HR, Heart rate; PPG, Photoplethysmography; SR, Step rate.
